# Polysaccharides Isolated from Açaí Fruit Induce Innate Immune Responses

**DOI:** 10.1371/journal.pone.0017301

**Published:** 2011-02-28

**Authors:** Jeff Holderness, Igor A. Schepetkin, Brett Freedman, Liliya N. Kirpotina, Mark T. Quinn, Jodi F. Hedges, Mark A. Jutila

**Affiliations:** Department of Immunology and Infectious Diseases, Montana State University, Bozeman, Montana, United States of America; Centre de Recherche Public de la Santé (CRP-Santé), Luxembourg

## Abstract

The Açaí (Acai) fruit is a popular nutritional supplement that purportedly enhances immune system function. These anecdotal claims are supported by limited studies describing immune responses to the Acai polyphenol fraction. Previously, we characterized γδ T cell responses to both polyphenol and polysaccharide fractions from several plant-derived nutritional supplements. Similar polyphenol and polysaccharide fractions are found in Acai fruit. Thus, we hypothesized that one or both of these fractions could activate γδ T cells. Contrary to previous reports, we did not identify agonist activity in the polyphenol fraction; however, the Acai polysaccharide fraction induced robust γδ T cell stimulatory activity in human, mouse, and bovine PBMC cultures. To characterize the immune response to Acai polysaccharides, we fractionated the crude polysaccharide preparation and tested these fractions for activity in human PBMC cultures. The largest Acai polysaccharides were the most active *in vitro* as indicated by activation of myeloid and γδ T cells. When delivered *in vivo*, Acai polysaccharide induced myeloid cell recruitment and IL-12 production. These results define innate immune responses induced by the polysaccharide component of Acai and have implications for the treatment of asthma and infectious disease.

## Introduction

Herbal products have been used in traditional medicines for a variety of infectious and inflammatory diseases. Many of these plant materials enhance the activity of cells of the innate immune system and modify host responses [1]–[5]. Though their use is widespread, in many instances the details of these immune responses are unknown and the optimal therapeutic potential of these materials is unrealized. Most studies describing the innate immune response to herbal products focus on cells of the myeloid lineage, such as macrophages and neutrophils. Our studies incorporate an additional cell type, the γδ T cell, which responds to at least three distinct herbal components [1], [3], [6]. γδ T cells are well placed to respond to traditional medicines as they are found in the intestinal mucosa and virtually all portals of entry in the body. These T cells contribute to effective innate immune responses against a variety of infectious agents [7]–[11]. They also facilitate downstream adaptive immune responses, due in part to cytokine production [12]–[14] and stimulation of dendritic cell function [15], [16]. γδ T cells are also potent cytolytic cells [17], [18], can present antigen [19], [20], induce or suppress inflammation [21]–[23], and are important to the health of epithelial cell monolayers [24], [25]. Due to the abundance of functional responses elicited by this cell type, its role in response to traditional medicine may have clinical relevance. In fact, novel therapeutic protocols, developed from natural agonists for the γδ T cell, are being pursued in the clinic to combat infection and cancers [26], [27].

The consumption of some traditional medicines is associated with the induction of γδ T cell activity. For example, the health benefits of tea consumption can be linked to stimulation of the human γδ T cell anti-microbial response [28], [29]. Additionally, some fruit and vegetable juices expand γδ T cells following consumption [30], [31]. Recently, we elaborated upon this association between traditional medicines and γδ T cell activity by defining immunomodulatory components within common traditional medicines.

Thus far, three functionally and structurally distinct plant-derived agonists capable of inducing γδ T cell responses are described: prenyl phosphates, polyphenols, and polysaccharides. Prenyl phosphates derived from plant and microbial sources [6], [32] rapidly expand a subset of human γδ T cells, but function only in primate species. The polyphenol agonists act directly on the γδ T cell, and other cell types, by inducing a priming response, typified by up-regulation of activation markers and an increased responsiveness to secondary stimuli [1]. While γδ T cell activating polyphenols are found in several plants [3], the polyphenol agonists are best illustrated with non-ripe apple peel polyphenols (APP) [1]. The most active polyphenol fraction identified consists of oligomeric procyanidins (OPCs) [3], however, other polyphenols including oenothein B contain agonist activity [33 and unpublished observations], suggesting polyphenol agonists are structurally heterogeneous. The final type of plant product with γδ T cell agonist activity includes polysaccharides from Yamoa™, the ground bark of the *Funtumia elastica* tree [1], [2]. Yamoa™ polysaccharides (referred to herein as Yam-1) induce *in vitro* effects on γδ T cells from bovine calves, humans, and mice. While γδ T cells respond directly to Yamoa polysaccharides, these responses are greatly amplified during monocyte/macrophage co-culture [2]. As a limitation to our understanding its activity, Yamoa™ exhibits reactivity in the limulus amebocyte lysate (LAL) assay. For this reason, separating polysaccharide agonist activity from the endotoxin activity in this preparation is problematic. Nonetheless, there is apparently an endotoxin-independent component of Yamoa™ as evidenced by a retained response in MyD88^−/−^ and TLR4^−/−^ mice [2].

The fruit from Acai, *Euterpe oleracea*, has become a popular nutritional supplement with anecdotal claims in support of immune stimulation. Since Acai contains both polyphenols and polysaccharides, it was tested for γδ T cell agonist activity. Whereas others have reported that the major polyphenol components from Acai contain immunomodulatory functions [34]–[38], we found that the polysaccharides, and not the polyphenols, from Acai stimulated bovine, mouse and human γδ T cells in mixed leukocyte cultures. Acai-derived polysaccharides also stimulated monocytes/macrophages, which was not due to endotoxin contamination, since the Acai fractions were found to contain very low LAL activity and were similarly active after polymyxin B treatment to remove residual endotoxin. Moreover, we evaluated proinflammatory responses to Acai polysaccharides *in vivo*; after either intraperitoneal or intratracheal administration of Acai polysaccharides, neutrophil recruitment to the respective tissue was observed. Furthermore, delivery of Acai polysaccharides to the lung activated alveolar macrophages and induced IL-12 production. Overall, these results indicate that the polysaccharide fraction is responsible, at least in part, for the immune responses reported for Acai and underscore its potential use as a therapeutic or prophylactic treatment for infectious disease.

## Materials and Methods

### Ethics Statement

All animal experiments were performed in accordance with National Institutes of Health guidelines and approved by the Institutional Animal Care and Use Committee (protocol identification: 2008-15, 2009-3). Human subjects testing was performed in accord with the Institutional Review Board of Montana State University (approval identification: MJ032609) and written, informed consent was obtained from all individuals.

### 2.1. Animals

TLR4^−/−^ and TCRα^−/−^ mice (both on the C57BL/6 background) as well as C3H-HeJ and C3H-HeOuJ mice used in this study were originally obtained from Jackson Laboratories (Bar Harbor, ME). MyD88^−/−^ mice were kindly provided by Dr. Kieren A. Marr. All mice were housed and bred at the Animal Resource Center at Montana State University.

One to four month-old bull Holstein calves used in this study were housed at the large animal facility of Montana State University.

### 2.2. Polysaccharide isolation from Acai

**Figure 1:**
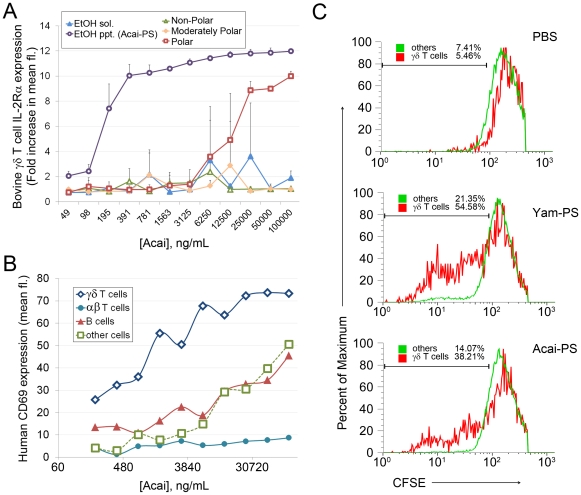
γδ T cell stimulatory activity in Acai is concentrated in the polysaccharide fraction and effective in all species tested. A) Aqueous extract of Acai was separated via EtOH precipitation or Kupchan fractionation. The resulting fractions were lyophilized, re-suspended in water, and tested in bovine PBMC culture for γδ T cell agonist activity. Data represent mean and SD from triplicate cultures from the same calf. EtOH precipitant (ppt.) responses are representative of cultures from three calves and three separate preparations. B) Human cells were cultured for 48 h with Acai-PS or medium prior to analysis for cell activation (CD69 expression) using flow cytometry. Values represent the average response of duplicate cultures from a single donor. Data are representative of two experiments. C) CFSE-labeled TCRα^−/−^ splenocytes were cultured in X-VIVO with PBS, Yam-PS (9 µg/mL), or Acai-PS (10 µg/mL) for 24 h, then medium was replaced with fresh medium containing IL-2 and cultured for an additional 72 h. Percent cell proliferation was determined as the percent of γδ T cells (lymphocyte, GL3^+^ gates) or others (lymphocyte, GL3- gates) divided at least once and are representative of two Acai-PS preparations.

Acai fruit pulp was obtained from two sources: Acai 100 (Genesis Today; Austin, TX) and Acai Berry Pure (Acai Berry Pure Bulk; Carlsbad, CA). The Acai 100 preparation consisted of 100% pure, liquid-format Acai fruit and was used to obtain preliminary results (data not shown) as well as to prepare the preliminary EtOH precipitation and Kupchan fractionation [39] products assayed in Figure 1A (prepared by contract: PhytoMyco Research Corporation; Greenville, North Carolina).

All other experiments utilized Acai Berry Pure. Polysaccharides were isolated from this powdered Acai as described previously for other polysaccharides[2]. Briefly, 1500 g of Acai powder was extracted with 8 L boiling distilled H_2_O for 1 h. The aqueous extract was then centrifuged at 2,000 x g for 15 min, and a 4-fold volume of ethanol was added to the supernatant to precipitate polysaccharides overnight at 4°C. The precipitate was pelleted by centrifugation, re-dissolved in distilled H_2_O, and centrifuged at 2,000 x g for 15 min. The supernatant fluid (crude polysaccharide extract) was fractionated using ion-exchange chromatography on a DEAE-cellulose column equilibrated with 0.05 M Tris-HCl buffer (pH 8.0). Bound material was sequentially eluted with 0.05 M Tris-HCl buffer and 2 M NaCl; a recovery of 0.27% by weight or 4 g total weight was achieved. The presence of polysaccharides in the unbound fraction, eluted with 0.05 M Tris-HCl buffer was minimal (<0.1% of total bound fraction). The Acai-PS fraction was generated from the bound material after concentration in an Amicon concentrator with a 10 kDa Amicon PM10 membrane (Millipore; Billerica, MA). Further fractions were produced by size exclusion chromatography on a Sepharose-6B column (2.5×95 cm) equilibrated with 0.01 M Tris-HCl buffer (pH 7.2) containing 0.15 M NaCl and eluted with the same buffer at a flow rate of 22 mL/h. The relevant fractions were pooled and concentrated. Three fractions were obtained, designated as Acai-1 (0.7 g total weight), Acai-2 (1.5 g total weight), and Acai-3 (0.85 g total weight). These fractions were analyzed by HPLC, and elution was monitored with a refractive index detector as described previously [40].

### 2.3. Polyphenol isolation and removal

Acai polyphenols were extracted from dried fruit pulp using the method described by Rodrigues *et al* [38]. Briefly, 100 g of Acai fruit (Acai Berry Pure) was washed over a three day period with exchanges of 350 mL, 350 mL, and 300 mL of MeOH. Next, the MeOH-extracted material was dried using a Savant SpeedVac® Plus SC210A Concentrator (Thermo Scientific; Waltham, MA). To isolate polyphenols, 5 g of polyvinylpolypyrrolidone (PVPP; Sigma-Aldrich, St. Louis, MO), triple-washed in water, was added to 5 mL of 24 mg/mL water-reconstituted, MeOH-extracted Acai. Polyphenols were allowed to adsorb to the PVPP matrix for 10 min prior to triple-washing with 20 mL DPBS. Polyphenols were eluted with 10 mL of 0.5 N NaOH for 5 min. The resulting polyphenols were adjusted to approximately pH 7.0 with HCl, dried to determine weight, and tested for their ability to stimulate human peripheral blood mononuclear cells (PBMCs).

For a second approach to measure polyphenol contribution, Acai-PS (100 mg) was transferred over a column containing 2 g of triple-washed PVPP. The eluent was filtered through a 0.2 µm filter and similarly assessed for stimulatory activity.

### 2.4. Characterization of Acai-PS fractions

The presence of type II arabinogalactan structures was detected by single radial diffusion in a 1% agarose gel containing 0.1 mg/mL β-glucosyl-Yariv reagent (4-β-d-glucopyranosyl oxyphenylazo-2,4,6-trihydroxybenzene [Biosupplies; Melbourne, Australia]) which specifically interacts with and precipitates compounds containing type II arabinogalactan structures. A solution of 2 mg/mL arabic gum (Fluka; St. Louis, MO) in H_2_O was used as a standard, and the polysaccharide samples were tested at 2 mg/mL. After application of 6 µL samples, the gels were incubated for 24 h at room temperature in a humid atmosphere. Arabinogalactan-positive reactions were identified by a reddish circle (halo) around the wells.

Fluorescence measurements were performed using an LS50 luminescence spectrometer (Perkin Elmer). Samples were dissolved in NaHCO_3_ (25 mM, pH 8.5). Synchronous fluorescence spectra were recorded from 300 to 600 nm at a scan rate of 240 nm/min. The excitation–emission wavelength difference (δλ) was 20 nm.

Protein content was measured using the Bradford method as per the manufacturer's directions (Bio-Rad Protein Assay: Bio-Rad; Hercules, CA). Bovine serum albumin was used to generate a standard curve. Absorbance was measured at 595 nm using a SpectraMax Plus microplate reader (Molecular Devices; Sunnyvale, CA).

The approximate molecular weight of the Acai-PS fractions was determined by high performance size exclusion chromatography (HP-SEC) using a Shimadzu Class VP HPLC and Shodex OHpak SB-804 HQ column (8 mm×300 mm) as previously described [41]. The molecular weights were estimated by comparison to the retention times of pullulan polymer standards (P-800, -400, -200, -100, -50, -20, and -10; Phenomenex, Torrance CA).

Monosaccharide analysis was performed by the Oklahoma Center for Glycobiology Analytical Core Lab (Oklahoma City, OK). Briefly, polysaccharide samples or background blanks were subjected to methanolysis (methanolic 2 M HCl, 16 h, 80°C), followed by acid hydrolysis (2 M trifluoroacetic acid, 2 h, 100°C), and the resulting monosaccharide mixtures were analyzed by high-performance anion-exchange chromatography with pulsed amperometric detection (HPAEC-PAD) on a Dionex DX-600 HPAEC system equipped with an ED50 detector (Dionex Corporation; Sunnyvale, CA). The samples were separated on a Dionex CarboPac PA-1 column eluted isocratically with 6 mM or 26 mM NaOH for 30 min, then a 100 mM NaOH gradient for 10 min followed by a sodium acetate gradient from 0 to 500 mM for 35 min at a flow rate of 1 mL/min at 22°C. For analysis of uronic acids, the column was eluted with 10 mM NaOH for 20 min, followed by a gradient of 100 mM NaOH/150 mM sodium acetate (0–100% for the duration of 45 min). Background signals were subtracted from all samples, and individual components were quantified based on electrochemical detection relative to known standards [42], [43].

Carbohydrate content was determined for Acai-PS by phenol-sulfuric acid method, modified to a microplate format [44], and absorbance was measured at 490 nm using a SpectraMax Plus microplate reader. A solution was prepared based on Acai-1 sugar content as: 4.5% L-rhamnose (Sigma-Aldrich), 47.0% L-(+)-Arabinose (Sigma-Aldrich), 11.5% D-(+)-galactose (Sigma-Aldrich), 2.8% D-(+)-xylose (Sigma-Aldrich), 28.4% D-(+)-galacturonic acid (Fluka), and 3.0% D-glucuronic acid (Sigma-Aldrich) by weight solution in DPBS. This solution was used to generate a standard curve.

The total amount of polyphenols in the Acai fractions was determined by Folin-Ciocalteu assay [45] as previously described [1]. Briefly, 250 µL of Folin's phenol reagent was added to the samples dissolved in 500 µL distilled water. After 3 min at room temperature, 1.25 mL of 20% sodium carbonate was added, mixed, and the mixture was allowed to stand for 40 min. The absorbance was measured at 750 nm in a spectrophotometer (DU800: Beckman Coulter; Brea, CA). The total polyphenol content was determined using epicatechin to generate a standard curve and expressed as epicatechin equivalents (epicatechin/mg sample)×100.

LAL assay was used to estimate the amount of endotoxin contained in the polysaccharide fractions from Acai. For all samples tested except the crude acai EtOH precipitation, a Pyrochrome LAL reagent reconstituted with Glucashield in an endotoxin-free microplate (all from Associates of Cape Cod; East Falmouth, MA) as per manufacturer's procedures was used. Analyses of endotoxin concentration were performed via the kinetic method using a VersaMax plate reader with SoftMax Pro software (Molecular Devices). The crude EtOH-precipitated Acai was tested for LAL reactivity using the PYROGENT (Cambrex; Charles City, IA) 0.125 EU/mL sensitivity inverted tube assay. To calculate the potential endotoxin content in Acai-1, Acai-2, and Acai-3 a ratio of 8 EU/ng was used.

### 2.5. Endotoxin removal

To remove potential contaminating endotoxin Acai-1 was applied to a column containing Detoxi-Gel Endotoxin Removing Gel (Pierce; St. Louis, MO) and eluted with 0.05 M phosphate buffer containing 0.5 M NaCl to decrease ionic interactions of sample molecules with the affinity ligand. The concentration of polysaccharides in the eluted sample (Acai-1^ER^) was adjusted to match that of the untreated fraction (Acai-1), as determined by diene group content (absorbance at 254 nm) [46].

### 2.6. Cell cultures

All cells were cultured at 37°C in a humidified atmosphere containing 5% CO_2_. Human monocyte-macrophage MonoMac-6 cells (DSMZ; Brunswick, Germany) were grown in RPMI 1640 (Mediatech Inc.; Herndon, VA) supplemented with 10% (v/v) FBS, 10 µg/mL bovine insulin, 100 µg/mL streptomycin, and 100 U/mL penicillin.

For primary cells, whole blood was collected from 1–3 month old bull Holstein calves into sodium heparin tubes (Becton Dickinson; Franklin Lakes, NJ) or healthy human adult donors with ACT tubes (Becton Dickinson). Peripheral blood mononuclear cells (PBMCs) were separated from whole blood using Histopaque 1077 (Sigma-Aldrich) for bovine cells as previously described [47] and human cells, as per the manufacturer's instructions. Additionally, bovine red blood cells were removed by hypotonic lysis. Preparation of spleens from TCRα^−/−^ mice for *in vitro* culture was performed as previously described [2]. Briefly, spleens were aseptically removed from the mouse, dounce homogenized, cold ACK buffer (8.29 g/L NH_4_Cl, 1 g/L KHCO_3_, 292 mg/L EDTA)-treated for 10 min, Nitex® filtered, and density separated using Lympholyte M (Cedarlane Laboratories; Burlington, NC) prior to culture at 2.5 E^6^/mL in XVIVO-15 medium (Lonza; Walkersville, MD). Splenocytes were infused with CFSE, cultured for 24 h with agonists, washed with fresh medium, and re-cultured for 72 h with medium containing recombinant murine IL-2 (rmIL-2 [Peprotech; Rocky Hill, NJ]).

### 2.7. Measurement of cell activation by flow cytometry

Flow cytometry was used to analyze cell activation in bovine, human, and mouse cultures as previously described [1], [3]. To measure activation, cells were stained with anti-γδTCR monoclonal Ab {GD3.8 (bovine [48]), GL3 (mouse; Becton Dickinson [49]), or 5A6.E9 (human; ATCC [50])} and either CD69 (human, FN50; Biolegend, San Diego, CA) or IL-2Rα/CD25 (bovine, LCTB2A [51]; VMRD, Pullman, WA). For human activation assays, anti-CD19 (HIB19, Biolegend) and -CD3 (UCHT1, Biolegend) were also used to identify lymphocyte populations as follows: αβT cells (CD3^+^, γδTCR^−^), B cells (CD3^−^, CD19^+^), γδT cells (CD3^+^, γδTCR^+^), other cells (CD3^−^, CD19^−^, γδTCR^−^).

To measure mouse splenocyte proliferation in response to rmIL-2, cells were stained with CFSE prior to culture as described [2] and then stained with anti- γδTCR mAb (GL-3) after culture. To differentiate mAb staining, FITC, PE, PE-Cy5.5, or Allophycocyanin (APC) fluorochromes were directly conjugated except for mAbs 5A6.E9 and LCTB2A, which were detected using fluorochrome-labeled goat-anti-mouse polyclonal Ab (Southern Biotech; Birmingham, AL). Indirect Ab staining was blocked using mouse serum before the addition of other Abs. Cells were analyzed using a FACSCalibur system equipped with a high-throughput sampler (Becton Dickinson). Results were analyzed using CellQuest Pro (Becton Dickinson) or FlowJo (Treestar; Ashland, OR) software.

### 2.8. Analysis of reactive oxygen species (ROS) production

ROS production was analyzed using the chemiluminescent probe, L-012, which is highly sensitive for ROS generated in biologically complex systems [52], [53]. Human PBMCs (2×10^5^ cells in 100 µL per well) were incubated with various concentrations of polysaccharide fractions or positive control LPS for 24 h. After incubation, culture supernatant fluid was replaced with an equal volume of HBSS supplemented with 25 µM L-012 as described previously. The reaction was monitored on a Fluoroscan Ascent FL microtiter plate reader (ThermoElectron; Milford, MA) at 37°C. Chemiluminescence was measured every 2 min for 3 h and is expressed as the integrated response over this time (arbitrary units).

### 2.9. Determination of Acai-induced cytokine production

Cells were incubated for 24 h in culture medium supplemented with 3% (v/v) endotoxin-free FBS, with or without Acai polysaccharide fractions or LPS as a positive control. Human PBMCs and MonoMac-6 human monocytic cells were plated in 96-well plates at a density 2×10^5^ cells in 100 µL per well. A human cytokine Multi-Analyte ELISArray™ Kit (SABiosciences Corporation; Frederick, MD) was utilized to evaluate various cytokines {interleukin (IL)-1α, IL-1β, IL-2, IL-4, IL-6, IL-8, IL-10, IL-12, IL-17A, interferon-γ (IFNγ), tumor necrosis factor α (TNF-α), and granulocyte-macrophage colony-stimulating factor (GM-CSF)} in supernatants of PBMCs. These results were confirmed using cells from a different donor with enzyme-linked immunosorbent assay (ELISA) kits for GM-CSF, TNF-α, IL-1β, IL-6, and IL-8 (all from Biolegend).

Human TNF-α or IL-6 enzyme-linked ELISA kits (Becton Dickinson and Biolegend) were used to quantify TNF-α or IL-6 levels in the cell supernatant fluids from PBMC or MonoMac-6 cultures.

### 2.10. Peritonitis assay

BALB/c mice were injected intraperitoneally (i.p.) with 5 µg Yam-1 [2], 5 µg or 50 µg of Acai-PS, or saline only. After 4 h, the mice were euthanized and the peritoneal cavity was washed with 10 mL HBSS (injected and retrieved) and cells collected. The number of neutrophils was quantified by mAb stain: CD45.2^+^ (104, Becton Dickinson), CD11b^+^ (M1/70, Becton Dickinson), and granulocyte receptor-1 (Gr-1;RB6-8C5^bright^ [54]). A known concentration of APC-labeled FACSbeads (Becton Dickinson) was added to these cells prior to flow cytometry using a FACSCalibur equipped with a high-throughput sampler (Becton Dickinson). Viable leukocytes were gated based on FSC/SSC and positive CD45 staining. The absolute count of neutrophils was calculated based on the number of beads collected versus the number of viable neutrophils and extrapolated for the 10 mL wash volume.

### 2.11. Lung inflammation assay

BALB/c mice (9–10 weeks, female) were instilled intratracheally (i.t.) with 1.56–500 µg Acai-PS 24 h prior to collection of bronchoalveolar lavage fluid (BALF) and lung tissue. Prior to tissue collection, mice were euthanized by CO_2_ asphyxiation. BALF was collected by lavage with two 1 mL washes of Hanks containing 2 mM EDTA. Approximately 1.5 mL lavage fluid was recovered from each wash. BALF was centrifuged and the supernatant fluid was saved for IL-12 ELISA (IL-12(p70); C15.6/C17.8-biotin, MabTech; Nacka Strand, Sweden). The pelleted BALF cells were treated for 10 min with cold ACK buffer to lyse red blood cells prior to analysis by flow cytometry. Next, lung tissue was collected by mincing with scissors then digestion for 1 h in collagenase/DNAse medium {200 U/mL collagenase (Worthington Biochemical; Lakewood, New Jersey) and 0.08 U/mL DNAse (Promega; Madison, Wisconsin) in RPMI with 20 mM HEPES} at 37°C. The resulting product was then passed through 35 µm Nitex® nylon mesh (Sefar America; Depew, NY) to remove tissue debris and ACK lysed. To analyze the cellular composition of the tissue and BALF, cells were stained with CD11b-FITC (M1/70; Becton Dickinson) and CD11c-APC (HL3; Becton Dickinson) prior to analysis using a FACS Calibur cytometer (Becton Dickinson).

### 2.12. Statistics

Statistical analyses were performed using Microsoft Excel or GraphPad Prism.

## Results

### Acai polysaccharides trigger minimal response in LAL detection assays and are potent agonists for bovine, human and mouse γδ T cells

In preliminary assays culturing peripheral PBMCs with crude Acai, we identified γδ T cell activation (up-regulation of CD69/IL-2Rα expression). This activity was independent of prenyl phosphates since bovine γδ T cells responded to this extract, suggesting the agonist activity could be due to polyphenols and/or polysaccharides [data not shown]. As a first step toward identification of the activating component(s) of the Acai extract, we separated the crude extract via Kupchan fractionation [39] or EtOH precipitation. Dose response assays were performed on the various fractions by measuring bovine γδ T cells activation in PBMC cultures. As shown in Figure 1A, EtOH-precipitated material induced γδ T cell activation as measured by IL-2Rα expression at low ng/mL concentrations. Furthermore, no activity was detected in the EtOH-soluble or non-polar fractions. Since polysaccharides precipitate in EtOH, whereas polyphenols, in general, remain soluble in ethanol, this suggested that polysaccharides were the agonist fraction in Acai. We next compared LAL reactivity in the EtOH-precipitated Acai to Yamoa™-derived polysaccharides (Yam-1) which are described in a previous report.^2^ EtOH-precipitated Acai had far less potential endotoxin reactivity than Yam-1 (>0.0000125 EU/ng for Acai versus 0.194 EU/ng for Yam-1). These experiments indicated that Acai polysaccharides activate γδ T cell populations without the potential endotoxin contamination or LAL cross-reactivity common to Yamoa™ and other polysaccharides.

To determine if Acai polysaccharides similarly activated mouse and human cells, we further purified the polysaccharides from EtOH-precipitated Acai using DEAE cellulose. This fraction, Acai-PS, was 92.1% polysaccharides as determined by phenol-sulfuric acid assay, and as expected, retained low LAL reactivity (0.0004 EU/ng). As shown in Figure 1B, Acai-PS increased the expression of CD69 on human lymphocytes, with the most robust activity detected in γδ T cells. Other cell populations including B cells and the unstained gate (other cells; presumably CD3^dim^ T cells, CD19^dim^ B cells, NK cells, and/or small monocytes) demonstrated increased CD69 expression to some degree. Mouse γδ T cells also responded to the Acai-PS fraction, as demonstrated by increased proliferation in response to rmIL-2 (Figure 1C). This *in vitro* priming response was repeated in TLR2^−/−^ and wildtype mice (C57BL/6). Importantly, we observed no toxic effects from Acai *in vitro* at concentrations up to 500 µg/mL [human PBMCs, data not shown] indicating these observed γδ T cell responses were not a result of cellular distress.

### γδ T cell agonist activity in Acai was found in the polysaccharide and not the polyphenol fraction

**Figure 2:**
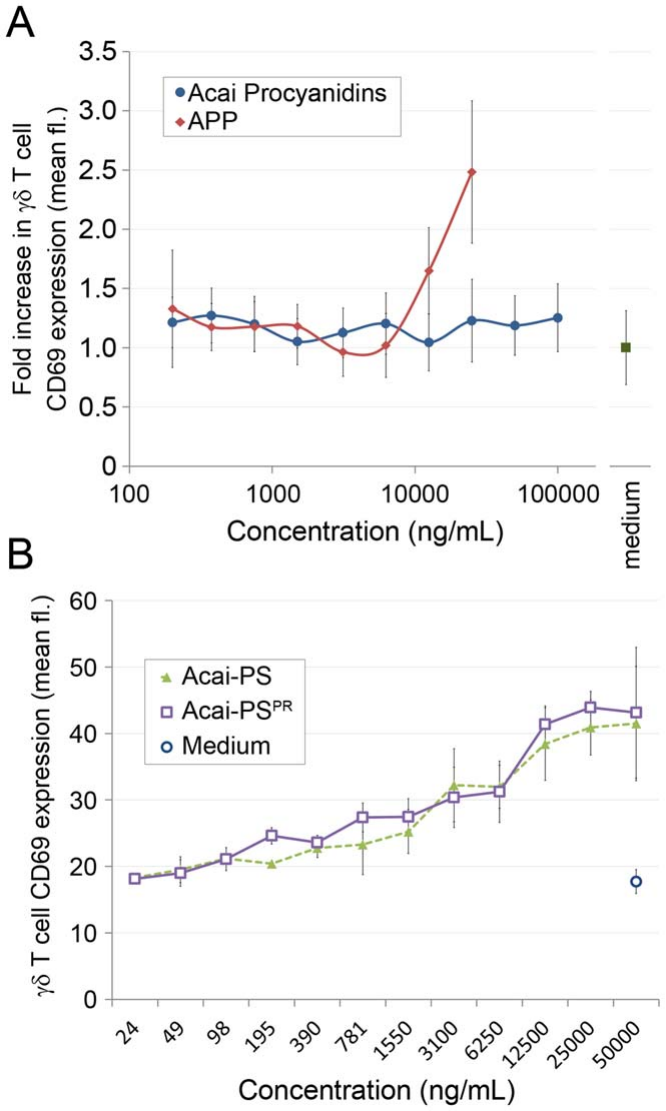
Acai fruit does not contain polyphenol-based γδ T cell agonists. A) PVPP-extracted Acai polyphenols were cultured with human PBMCs to detect γδ T cell activation. As a control, APP [1] was used to induce polyphenol-based γδ T cell activation. B) Acai-PS was treated with PVPP to remove polyphenols and the resulting preparation (Acai–PS^PR^) or untreated Acai-PS was cultured with human PBMCs. γδ T cell activation from the subsequent cultures was measured by FACS as induced CD69 expression. Results are from three individual donors. Error bars represent SD. Experiments were performed independently with respect to donor, experiment date, and Acai-PS^PR^ extraction.

Since earlier reports demonstrated immunomodulatory activity of Acai polyphenols [34]–[36] and we have found that some polyphenols are potent γδ T cell agonists [1], [3], we purified polyphenols from Acai and tested them for activity. As shown in Figure 2A, purified Acai polyphenols did not increase CD69 expression on γδ T cells at dosages nearly ten times higher than the polyphenol agonist, Apple Polyphenol (APP) [1]. Also, there was no shift in the dose response curve of Acai-PS in which polyphenols were removed (Acai-PS^PR^) by PVPP pre-treatment (Figure 2B). These results indicate that the previous reports describing polyphenol-induced immune responses were not a result of activated γδ T cells.

**Figure 3:**
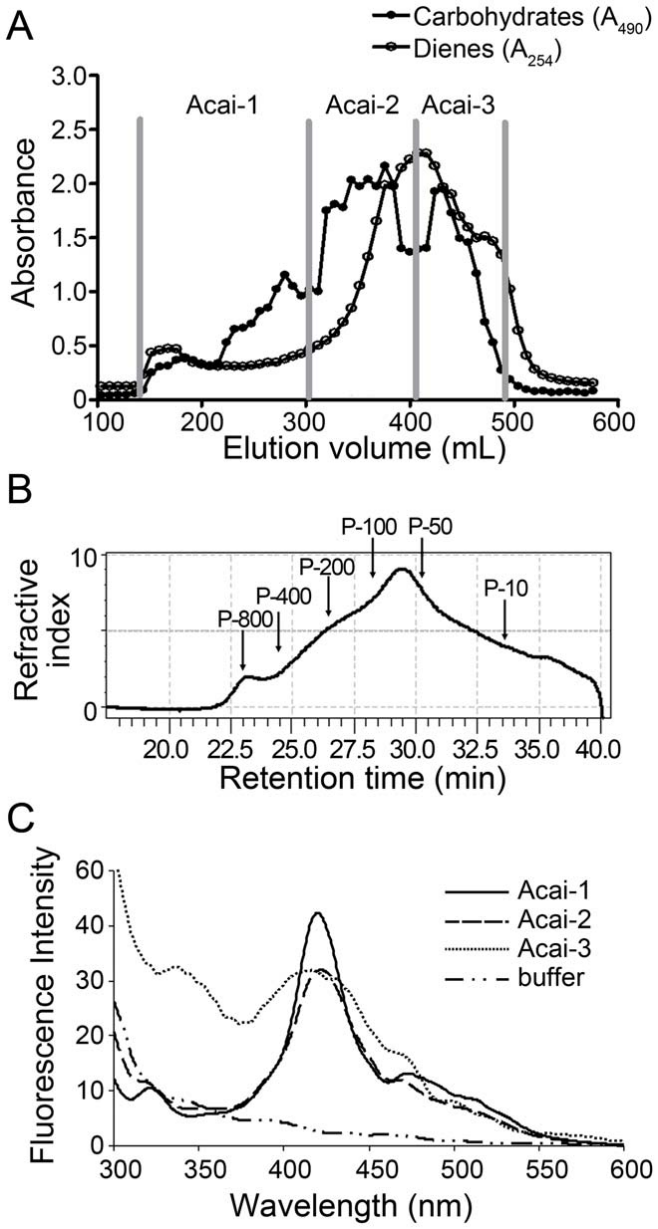
Chromatographic characterization and fractionation of Acai polysaccharides. Water extract of Acai was prepared and separated on DEAE-cellulose column (Acai-PS) and quantified using multiple methods: A) Acai-PS fractionation by gel chromatography on Sepharose-6B column. Three polysaccharide fractions (designated Acai-1, Acai-2, and Acai-3) were selected based on total carbohydrate and diene conjugate contents. B) High pressure gel filtration chromatography elution profile of Acai-PS with a refractive index detector. C) Synchronous fluorescence spectra of polysaccharides isolated from Acai-PS{500 µg/ml of each polysaccharide fraction in NaHCO_3_ buffer (pH 8.5)}.

To better characterize the Acai polysaccharide agonist, the Acai-PS extract was then fractionated by preparative Sepharose 6B size-exclusion chromatography to obtain three fractions, which were selected based on the total carbohydrate elution profile (designated as: Acai-1, Acai-2, and Acai-3, Figure 3A). Based on calibration curves derived from pullulan standards [41], we determined that fraction Acai-1 was composed of molecules with mass ∼200,000 Da as well as a small sub-peak at ∼800,000 Da, which could represent molecular aggregates. Acai-2 had a broad peak at ∼26,000–60,000 Da, and Acai-3 contained a broad peak at 4,000–12,000 Da (Table 1). As expected, all of these fractions remained low in LAL reactivity (Table 1). The fractions were then analyzed for polysaccharide and protein content and found to contain >99% carbohydrate and <1.0% protein (Table 1). Note that the carbohydrate profile was in accord with that of refractive index chromatogram obtained by HP-SEC (Figure 3B).

**Table 1:** Biochemical and spectral properties of Acai polysaccharide fractions.

Polysaccharide fraction	Average molecular weight (kDa)	Potential endotoxin (ng/µg)	Protein content (w/w)	Total phenolic content (w/w)	Type II arabinogalactan
Acai-1	200	0.33	0.35%	0.2%	Positive
Acai-2	26–60	0.05	0.43%	2.8%	Positive
Acai-3	4–12	0.01	0.60%	5.9%	Positive

All three fractions exhibited fluorescence emission in the broad region of 370–540 nm (Figure 3C). This finding suggested the presence of aromatic groups in context of the polysaccharides; however, this method will detect minute levels of aromatic groups, therefore the relative contribution of aromatics could not be estimated from this assay. To estimate potential aromatics, we performed a Folin-Ciocalteu assay. Results indicated polyphenols were a trivial component of the Acai-1 fraction (0.2%) and a minor component of the Acai-2 and Acai-3 fractions, 2.8% and 5.9%, respectively (Table 1). This result further demonstrated that the polysaccharide and not the polyphenol fraction was the predominant source of γδ T cell activity in the Acai preparation.

Very-high-field (600 MHz) ^1^H NMR was used to characterize the structure of the native Acai polysaccharides. The spectra from all three fractions (Figure S1) suggested a backbone structure resembling native arabinogalactans isolated from various plant sources [55], [56]. Using evaluation methods previously described for the sugar composition of the arabino-3,6-galactans (type II) [55], [56], we identified the presence of unsaturated α-rhamnopyranose, β-galactopyranose, α-arabinofuranose, and α-galacturonopyranose. All spectra also indicated a significant amount of N- and O-acetyl (1.9–2.0 ppm), methyl (0.75 and 1.1 ppm), and alkylamide (3.21 ppm) groups.

Type II arabinogalactan contains a β-(1,3)-linked galactan backbone with side chains containing arabinose and galactose residues and has reported biological activities in other systems [57], [58], but importantly, polysaccharide fractions containing type II arabinogalactan also contain the most activity as γδ T cell agonists [2,41 and unpublished results]. To evaluate the Acai polysaccharide fractions for arabinogalactan, the Yariv test was performed. All three fractions resulted in a positive reaction, indicating they contained arabinogalactan type II (Table 1). Sugar composition analysis revealed that the Acai polysaccharides consisted primarily of arabinose, galacturonic acid, and galactose (Table 2) which supports the results of the Yariv test and the presence of arabinogalactans in these fractions.

**Table 2:** Monosaccharide composition of Acai polysaccharide fractions.

	Polysaccharide fraction		
Monosaccharide	Acai-1	Acai-2	Acai-3
Fucose	0.5	0.6	1.3
Rhamnose	4.5	4.1	4.9
Arabinose	47.0	26.2	18.8
Glucosamine	N.D.	N.D.	N.D.
Galactose	11.5	17.9	16.5
Glucose	2.3	10.4	18.8
Lyxose	N.D.	N.D.	N.D.
Mannose	N.D.	4.7	8.4
Xylose	2.8	9.7	8.0
Galacturonic acid	28.4	24.7	21.7
Glucuronic acid	3.0	1.8	1.5

The data are present as mol% for each sugar. Individual components were identified and quantified based on elution of known standards. N.D. – not detected.

These results indicated that the Acai polysaccharide fractions Acai-1, Acai-2, Acai-3 contain different structures as determined by size, sugar content, and NMR. Furthermore, these fractions were predominantly polysaccharide with very low polyphenol or protein content.

### Acai polysaccharides induce γδ T cell and myeloid cell activation

**Figure 4:**
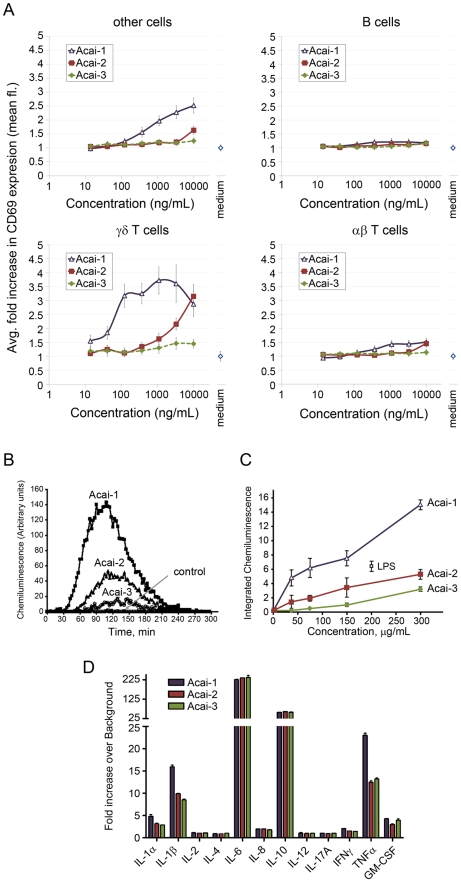
Acai fractions induce cell activation, as well as, ROS and cytokine production. A) PBMCs were collected from three donors and cultured with indicated agonists at various concentrations (x axis). Cultures were performed in triplicate. Data represent the mean fold increase (CD69 mean fluorescence) versus medium for each agonist/concentration value. Error bars represent normalized SD. B) PBMCs were incubated with polysaccharide fractions (150 µg/mL) and ROS production was measured over 300 min. C) ROS production from PBMCs was measured as a function of dose. PBMCs were incubated with the indicated concentrations of polysaccharide fractions, LPS, or vehicle only for 24 h. ROS production was then measured for 3 h from triplicate samples. Data represent the mean ± SD total luminescence over 3 h. Values are from one experiment, representative of three independent experiments. D). An ELISA was used to measure cytokine production by human PBMCs treated with 50 µg Acai-PS. Values represent the mean fold increase versus medium control cultures from triplicate wells. Error bars represent SD. Cultures were from one subject. Production of IL-1β, IL-6, GM-CSF, and TNF-α, and as well as limited IL-8 was confirmed in PBMCs from at least one additional donor using different ELISA reagents.

The activity of the Acai-PS fractions was tested using the CD69 expression assay for human PBMC cultures. As shown in Figure 4A, treatment with Acai-1 and to a lesser extent Acai-2 activated human γδ T cells with minimal activity on αβ T cells and B cells. We also tested the monomeric sugars from Acai-1. These sugars had no agonist effect [data not shown], indicating the complex structure of Acai-PS is important to its immune activity.

Since other polysaccharide preparations are potent inducers of reactive oxygen species (ROS) formation [41], we tested Acai polysaccharides for similar responses. In the absence of any treatment, human PBMCs did not generate detectable ROS (Figure 4B, control), whereas the addition of Acai-PS fractions activated ROS production with a lag-phase of around 30 min. A concentration-dependent enhancement of ROS production was observed in PBMCs treated with 37.5–300 µg/mL of each polysaccharide fraction (Figure 4C). As in the γδ T cell activation assays, Acai-1 demonstrated the most activity.

We next examined cytokine production by Acai-treated human PBMCs. Among the twelve cytokines analyzed, six were consistently induced in PBMCs by 100 µg/mL of Acai polysaccharide fractions, as compared with control cells. For Acai-1, these included IL-1α {fold increase (FI) = 4.8}, IL-1β (FI = 15.9), IL-6 (FI = 223), IL-10 (FI = 57), TNF-α (FI = 23), GM-CSF (FI = 4.2) (Figure 4D).

**Figure 5:**
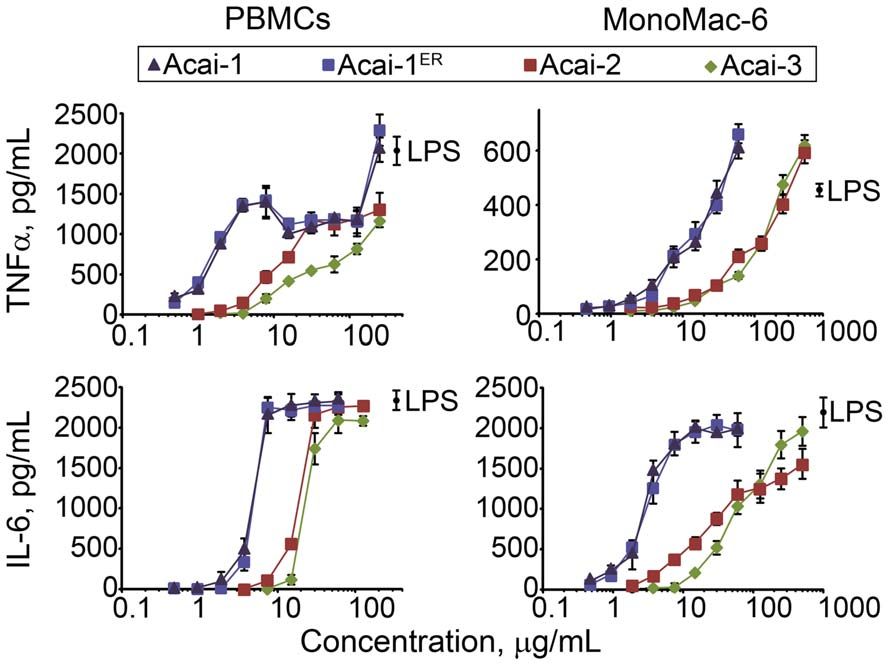
Effect of Acai polysaccharide on TNF-α and IL-6 production in MonoMac-6 and human PBMCs. Human PBMCs or MonoMac-6 macrophages were incubated for 24 h with the indicated concentrations of polysaccharide fractions Acai-1, Acai-1 pretreated with endotoxin-removing gel (Acai-1^ER^), Acai-2, Acai-3, or 200 ng/mL LPS. Cell-free supernatants were collected, and extracellular TNF-α and IL-6 were quantified by ELISA. Values represent the mean ± SD of triplicate samples from one experiment, which is representative of at least three independent experiments.

The ROS formation and cytokine secretion profile indicated that Acai polysaccharides function similarly to polysaccharide preparations active on monocytes [2], [41]. Therefore, we further analyzed the monocyte response to Acai polysaccharides. Although the amount of endotoxin (LPS) was very low and far larger amounts are required to activate γδ T cells [2], [59], monocytes are exquisitely sensitive to endotoxin. Thus, further steps were taken to ensure that endotoxin was not a component of the Acai preparation. To this end, we prepared a detoxified Acai-1 by elution through a column of endotoxin-removing gel (denoted Acai-1^ER^). To quantify dose-dependent effects of Acai polysaccharides on monocyte-associated cytokine production, levels of TNF-α and IL-6 were determined in cells treated with the polysaccharide fractions by ELISA. As shown in Figure 5, incubation of PBMCs with the fractions enhanced TNF-α and IL-6 production in a dose-dependent manner. Acai-1 and Acai-1^ER^ induced very similar responses indicating the minor endotoxin contamination had no effect on the activity of Acai-1. Furthermore, as with previous experiments, the Acai-1 fractions were the most active fraction at low concentrations (<1 µg/mL). Although a slight decrease in TNF-α production in Acai-1-treated cultures from 5 to 10 µg/mL was noted, the level of the cytokine was increased when cells were exposed to a higher dose (100 µg/mL) of Acai-1. We hypothesized this was due to the inherent heterogeneous nature of cell populations in PBMC cell preparations and their differing responses to the polysaccharides.

To address monocyte-specific responses to Acai polysaccharides, we tested the fractions on a human monocyte cell line, MonoMac-6. As with the PBMC cultures, Acai-1^ER^ induced nearly identical responses to the non-endotoxin-cleared Acai-1 in MonoMac-6 cells (Figure 5). MonoMac-6 cells also responded similarly to human PBMCs (Figure 5) without the cytokine secretion plateau, confirming that monocytes respond in a dose-dependent manner to Acai polysaccharides. The elimination of the cytokine secretion plateau and the greatly reduced TNF-α production in the MonoMac-6 cultures indicated monocytes were not the sole responding factor to Acai polysaccharides. These results, in combination with the activation of γδ T cells, were consistent with our earlier studies on Yamoa that indicate γδ T cells, monocytes, and possibly other cells cooperate for innate responses induced by polysaccharides [2].

### Acai polysaccharides induce immune recruitment and activation responses *in vivo*

**Figure 6:**
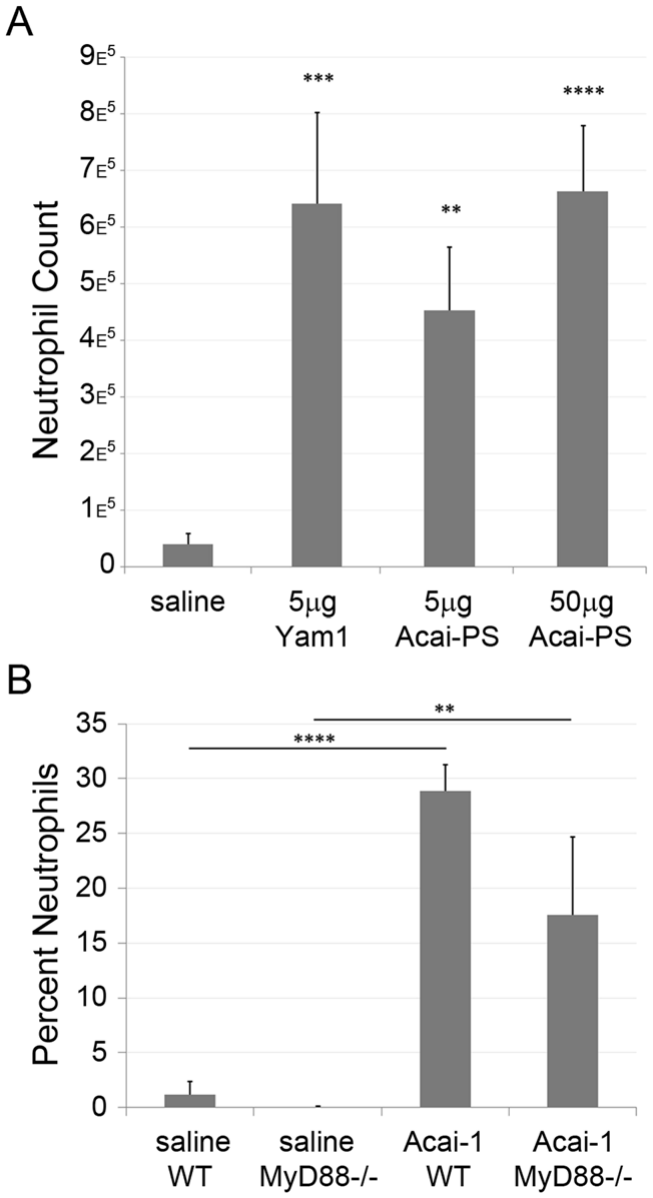
Acai polysaccharides induce MyD88-independent neutrophil influx to the peritoneum. A) BALB/c mice were injected intraperitoneally with saline, Acai, or Yam-1. After 4 h, mice were euthanized, peritoneal cells collected, and total neutrophil counts measured by flow cytometry. Data represent the average total cell count from a minimum of four mice per treatment group and error bars represent the SEM. B) C57BL/6 or MyD88^−/−^ mice of mixed ages (12–23 weeks) and sexes were injected i.p with Acai-PS (400 µg) or saline and neutrophil flux was measured as in A) without the use of FACS beads to estimate total cell counts. The data are representative of the mean percentage of neutrophils in the wash ± SD from a single experiment with 3–4 mice/group. p-values (Student's T test) for both figures are represented as: *<0.05, **<0.01, ***<0.005, ****<0.001.

Since Acai polysaccharides affect multiple cell types, we next sought to examine the combined effects of Acai-derived polysaccharides *in vivo*. To this end, we first examined their effect on the recruitment of neutrophils after intraperitoneal (i.p.) injection in mice. As shown in Figure 6A, Acai-PS induced neutrophil recruitment into the peritoneum, similar to LAL^+^ polysaccharides, Yam-1. Consistent with our previous report demonstrating a clear difference between Yam-1- and LPS-induced responses [2], the effect was not contingent upon MyD88 signaling (Figure 6B). Since MyD88^−/−^ mice are documented to possess an atypical immune response [60], [61], no conclusions can be reliably drawn from the apparent reduction of peritonitis in MyD88^−/−^ mice versus wild-type mice; it may be that there is a MyD88-dependent component to the full response or it may be due to strain differences. These results indicate that the *in vitro* immunostimulatory responses we observed toward Acai polysaccharides were preserved *in vivo*.

**Figure 7:**
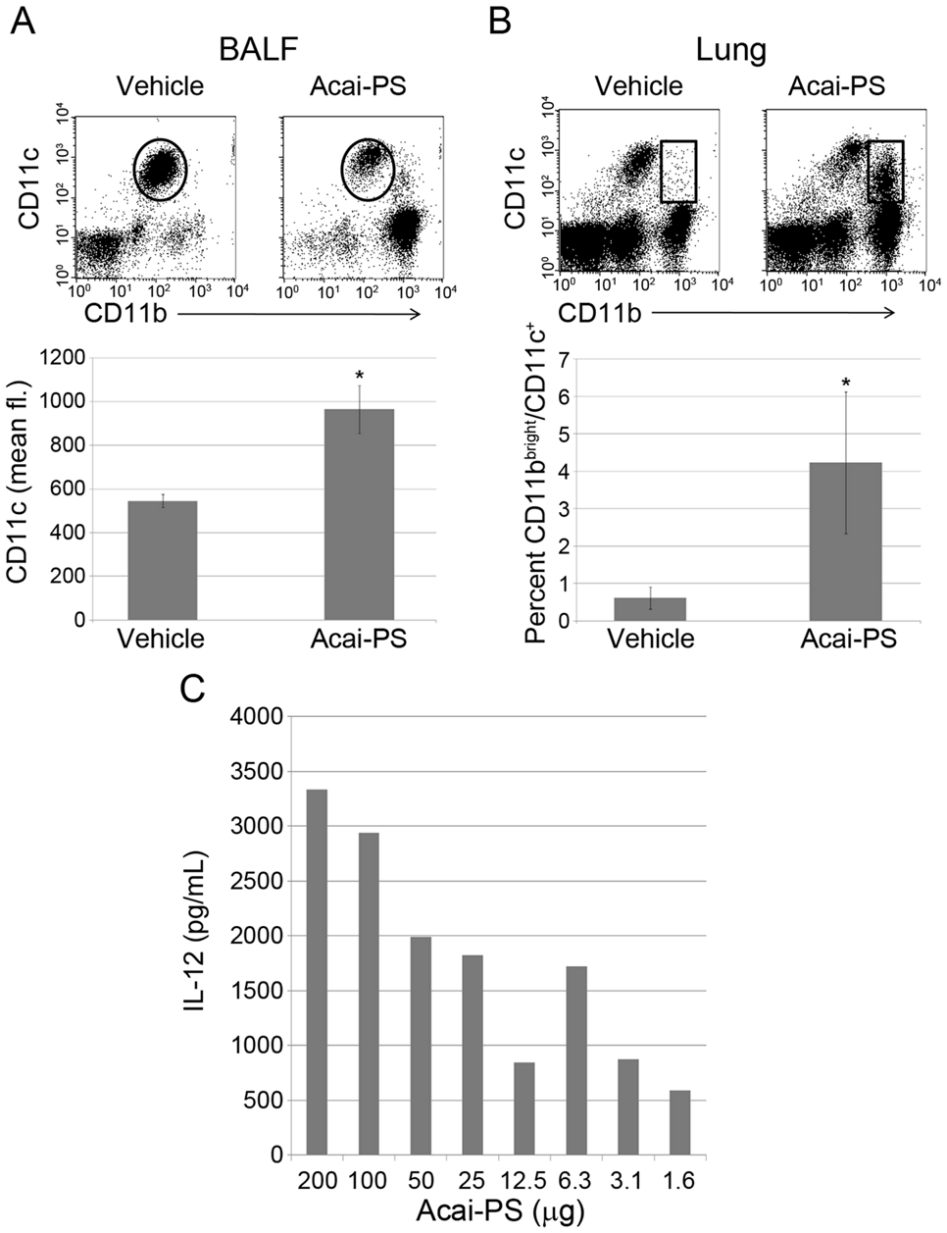
Intratracheal (i.t.) treatment with Acai-PS activates lung myeloid cells and induces IL-12 production in mice. BALB/c mice (n = 3) were treated i.t. with vehicle (dH_2_0) or 500 µg Acai-PS in a volume of 100 µL. BALF and lung cells were isolated 24 h post-treatment. Cells were stained with antibodies for CD11b and CD11c and analyzed via flow cytometry for myeloid cell activation/recruitment. A) BALF alveolar macrophages were gated (autofluorescent/CD11c^+^; ovals) and activation was measured as an increase in mean CD11c-associated fluorescence within this gate. B) Cells in the lung interstitium were collected via collagenase extraction and similarly analyzed by FACS for myeloid cell recruitment/activation. The percentage of myeloid cells (rectangle gate) in relation to total live leukocytes was compared between Acai-PS and vehicle treated mice. Data from A) and B) are representative of three similar experiments and were repeated in C57BL/6 (3 experiments) and C3H/HeOuJ (2 experiments). C) BALF was collected from BALB/c mice provided varying dosages of Acai-PS i.t. Cells were removed by centrifugation, and IL-12(p70) concentration was determined in the supernatant fluid by cytokine ELISA.

To determine if Acai-derived polysaccharides induce immune responses at mucosal surfaces, mice were treated i.t. with 500 µg Acai-PS and, 24 hrs later, cells in the BALF and lung tissue were extracted to measure myeloid cell activation/recruitment. In the BALF, alveolar macrophages (autofluorescent, Oval gate) increased expression of CD11c (Figure 7A), indicating these resident cells were activated. Similar to the peritonitis experiments, there was also a neutrophil influx detected in the BALF as shown by the increased CD11b^+^/CD11c^−^ population (Figure 7A). These cells were likely neutrophils because of their high Gr-1 expression and distinctive light scatter profiles [data not shown]. The remaining lung tissue was then homogenized and collagenase digested to collect the lung interstitial population. Flow cytometry detected an additional CD11b/CD11c positive cell population (Figure 7B, rectangles). It is unknown from these experiments whether these additional myeloid cells were recruited or whether they were activated resident cells. Regardless of the source of these activated myeloid cells, these experiments demonstrate a change in lung innate immune cell profile upon Acai administration.

To further characterize the lung response to Acai, the BALF was tested for the proinflammatory cytokine, IL-12. In initial studies from four mouse strains (BALB/c, C57BL/6, C3H/HeJ, and MyD88^−/−^) we observed an increased IL-12 content in Acai-PS-treated versus control animals [n≥2 for each strain, data not shown]. Therefore, to estimate the dose response, BALB/c mice were treated i.t. with a range of Acai-PS doses, and the concentration of IL-12 in the BALF was measured. As shown in Figure 7C, Acai-PS dose-dependently induced IL-12 production within the lung, indicating a proinflammatory T_H_1 response. Thus, the myeloid cell-associated response to Acai polysaccharides was conserved in both lung and peritoneal tissues. In contrast to the current thought that Acai polyphenols are responsible for immune enhancement [35], [36], [62], these data demonstrate immunostimulatory properties of Acai polysaccharides both *in vitro* and *in vivo*.

## Discussion

Although Acai is heavily marketed and currently taken by the general public to enhance immune cell function (presumably through antioxidant function), for weight loss, and for a variety of other unfounded claims, there have been few studies on its specific mechanisms of action. Indeed, much of the information justifying its use is anecdotal. Herein, we show that polysaccharides, but not polyphenols, derived from Acai fruit pulp have potent immunomodulatory activity and stimulated both γδ T cells and myeloid cells. The *in vitro* response to Acai-PS was conserved between mouse, bovine, and human cells and correlated with *in vivo* responses. In mice, Acai-PS incited neutrophil recruitment to the peritoneum and lung as well as activated DCs/macrophages in the lung. This peritonitis response occurred independent of MyD88 signaling, though at a lower level. This latter result, in combination with the minimal LAL reactivity, shows that bacterial-derived endotoxin is likely not responsible for the activity of Acai-derived polysaccharides and that these polysaccharides contain a distinct innate immune agonist.

The limited analyses reported to date suggest that polyphenols represent the immunomodulatory compounds in Acai [34], [35], [37], [38]. In contrast, the activity reported here tracked solely to the polysaccharide fraction of the Acai fruit pulp, and we found little to no activity from the polyphenols within this extract. Evidence against polyphenol-induced γδ T cell activation was based on: 1) an absence of immune cell activity in polyphenols purified from Acai-extract (Figure 2A), 2) a retained bioactivity in Acai preparations depleted of polyphenols (Figure 2B), and 3) the limited amounts of polyphenols in the purified Acai-fractions (Table 1). It is unlikely that the very small amounts of free polyphenols not removed by PVPP could account for our results, since a defining characteristic of γδ T cell immunomodulatory polyphenols is the relatively high concentrations (low µg/mL) required to induce cellular responses *in vitro* [1], [3]. It remains possible that polyphenol-complexed polysaccharides are required for biological activity since there was a small amount of polyphenols in the Acai-1, Acai-2 and Acai-3 fractions. However, the most active fraction, Acai-1, had the least amount of polyphenol (0.2%, Table 2), rendering this theory unlikely. It is therefore likely that the previous reports describing polyphenol activity were a result of general antioxidant effects. This is not unexpected since Acai has a very high antioxidant capacity [36], [63].

The fractions tested herein were derived from the Acai fruit pulp since it is the primary source of nutritional supplements and foodstuffs. The fruit pulp contains a relatively low concentration of the preeminent γδ T cell polyphenol agonist, OPC [62], [63], which could explain the lack of γδ T cell agonist activity. However, the seed from Acai fruit contains an enriched OPC profile very similar to APP or grape seed [38]. Since OPCs from APP, grape seed, and others activate γδ T cells [1], [3], [64], polyphenols extracted from Acai seeds may have similar effects on γδ T cells. While identifying the potential γδ T cell agonist activity of the polyphenol extract from Acai seed was beyond the scope of these studies, additional studies are underway to determine its biological activity. If it holds true that Acai seed polyphenols contain γδ T cell agonist activity, the seed could be prepared as a distinct nutritional supplement. Acai seeds are currently a byproduct of the Acai fruit, and are generally wasted or being used as pig feed or potting soil [38]. This seed may be an alternative and economically feasible source for γδ T cell-activating polyphenols for human applications.

Questions have been raised about the role of microbial contaminants in plant-derived products contributing to immunomodulatory activity [65], [66], thereby necessitating strict control over potential contaminants. Furthermore, products such as polysaccharides are particularly difficult to control for since they can cause false-positive LAL reactions [67], [68] and are difficult to isolate from endotoxin using conventional methods [2]. We found no evidence of microbial contamination in the Acai extract, as evidenced by minimal LAL reactivity and negative results of attempted bacterial culture [data not shown]. Furthermore, the bioactivity in Acai-1 could not be removed by polymyxin B (Figure 5), and activity occurred in animals deficient in MyD88 signaling (Figure 6B). Thus, sensing of microbial products through TLRs likely does not account for the innate cell responses shown in this report. However, it could very well be that MyD88-independent, yet TLR-dependent, pathways, such as TLR4-mediated TRIF signaling, are involved in recognition of the polysaccharides and these issues are currently under study. Furthermore, elaboration of processed IL-1β suggests that Acai polysaccharides could affect the inflammasome, for which agonists are highly variable [69], [70]. This possibility clearly warrants, and is under, further investigation.

To date, we have defined myeloid cell agonist activity in a number of plant extracts, including extracts from juniper berries [41], Artemisia [40], prickly-pear cactus [71], Yamoa™ [2], and now Acai. Activity on γδ T cells has also been observed with these extracts, excluding cactus [2, this report, and unpublished results]. Thus, the relevant γδ T cell agonists may be polysaccharides common to many plants. As such, we predict that other plants contain bioactivity similar to that in Acai, which may account for the expansion of γδ T cells in people that have consumed certain fruit and vegetable extracts [31].

As discussed in Graff *et al*., Yamoa™ is purported to be beneficial in asthma [2]. Asthma is associated with an exaggerated T_H_2 cytokine response mediated in part by γδ T cells. In mice, lung γδ T cells are present that can either promote or restrict T_H_2 cytokine responses [72]. Clinical evidence indicates that γδ T cells are increased [73] in asthmatic patients and also that these cells produce large amounts of T_H_2 cytokines after antigen challenge [74]. Since therapies to increase T_H_1 responses can alleviate asthma symptoms [75], we originally proposed that the anecdotal asthma benefits attributed to plant polysaccharides, might be a result of tipping the γδ T cell cytokine balance in the lung towards a T_H_1 response. At the time, we had no direct evidence in support of this hypothesis, and results would have been difficult to interpret due to the endotoxin-reactive component of Yamoa polysaccharides. Here we found that Acai-1 directly induced IL-12 production in the mouse lung. IL-12 release favors a downstream T_H_1 response via IFN-γ production from leukocytes in the mucosa [76]. Thus, we provide, for the first time, mechanistic evidence for the potential benefit of some plant polysaccharides by driving T_H_1 responses in the lung. In addition, IFN-γ is crucial for host defense responses against intracellular bacterial pathogens of the lung, such as *Francisella tularensis* [77] and *Coxiella burnetii* [78]. Efforts are currently underway to test the effectiveness of Acai polysaccharides in countering lung infections in these and a variety of other pulmonary infection models.

Another issue currently under investigation is whether the plant polysaccharide-induced immune cell activity can also be produced following oral ingestion. There are many variables within these experiments such as the effects of gastric enzymes, low pH, normal bacterial flora, and agonists in a normal diet on the agonist activity. However, Acai polysaccharide extracts are certainly resistant to harsh chemical conditions similar to the stomach, including high heat (boiling; see preparation in materials and methods) and low pH (1 M HCl; data not shown). To date, it is uncertain whether Acai polysaccharides translocate across epithelial barriers; however, there is precedence for modulation of systemic immune activity by consumption of plant- and microbial-derived glucans [79] as well as large polysaccharide polymers from *Aureobasidium pullulans* [80] and *Ganoderma lucidum* [81], [82]. Furthermore, immunomodulatory polysaccharides can impact intestinal leukocytes and enterocytes [83]. In the event that the polysaccharides described herein are restricted to acting upon cells within the intestine, intraepithelial γδ T cells could still be targeted. Potential therapeutic applications for a gut-restricted γδ T cell agonist include epithelial healing [24], [25] and improved immune responses to a variety of pathogen-associated diseases [16]. In preliminary studies, some mice administered Acai-PS or Yam-1 by oral gavage produced a cytokine response that could be detected within the serum [unpublished observations]. Therefore, it would seem that Acai polysaccharides are capable of inducing a systemic immune response. These preliminary results are being investigated in detail as part of ongoing studies to determine the *in vivo* and therapeutic potential for γδ T cell-agonist polysaccharides.

In closing, characterization of the immune responses or lack thereof for common nutritional supplements is important for potentially isolating new drug candidates but also for preventing potential misuse by the public. Herein, we define potent immunomodulatory activity from Acai on monocyte and γδ T cell populations. Unlike previous reports describing activity in the polyphenol fraction, we instead identified activity in the polysaccharide fraction. These polysaccharides were able to induce cell recruitment and T_H_1 responses *in vivo*. As such, potential applications for these polysaccharides include asthma and infectious disease. The absence of significant LAL reactivity is critical for the description of polysaccharide-induced immune responses *in vivo* and provides a readily available source for the development of a clinical preparation.

## Supporting Information

Figure S1 **^1^H NMR spectra of Acai-PS fractions.** The fractions were dissolved in D_2_O, and spectra were recorded at 20°C, as described [41]. Using previously described methods, we predict the following peak associations which are marked on the Acai-3 graph: weak signals present at 3.37–3.45 ppm represent α-rhamnopyranose (α-Rha *p*), the strong signals at 3.54–3.96 ppm represent β-galactopyranose (β-Gal *p*) [84], and the signals at 4.04–5.07 ppm represent α-arabinofuranose (α-Ara *f*) as well as α-galacturonopyranose (α-GalA *p*) residues [56], [84]. N- and O-acetyl (1.9–2.0 ppm), methyl (0.75 and 1.1 ppm), and alkylamide (3.21 ppm) groups are also represented.
(DOCX)
