# Supplementary material for: Polysaccharides Isolated from Açaí Fruit Induce Innate Immune Responses
Source: PLoS One. 2011 Feb 28;6(2):e17301. doi: 10.1371/journal.pone.0017301 (PMC3046208; doi:10.1371/journal.pone.0017301)
Supplement: Figure S1 — 1H NMR spectra of Acai-PS fractions. The fractions were dissolved in D2O, and spectra were recorded at 20°C, as described [41]. Using previously described methods, we predict the following peak associations which are marked on the Acai-3 graph: weak signals present at 3.37–3.45 ppm represent α-rhamnopyranose (α-Rha p), the strong signals at 3.54–3.96 ppm represent β-galactopyranose (β-Gal p) [84], and the signals at 4.04–5.07 ppm represent α-arabinofuranose (α-Ara f) as well as α-galacturonopyranose (α-GalA p) residues [56], [84]. N- and O-acetyl (1.9–2.0 ppm), methyl (0.75 and 1.1 ppm), and alkylamide (3.21 ppm) groups are also represented. (DOCX) [file pone.0017301.s001.docx]

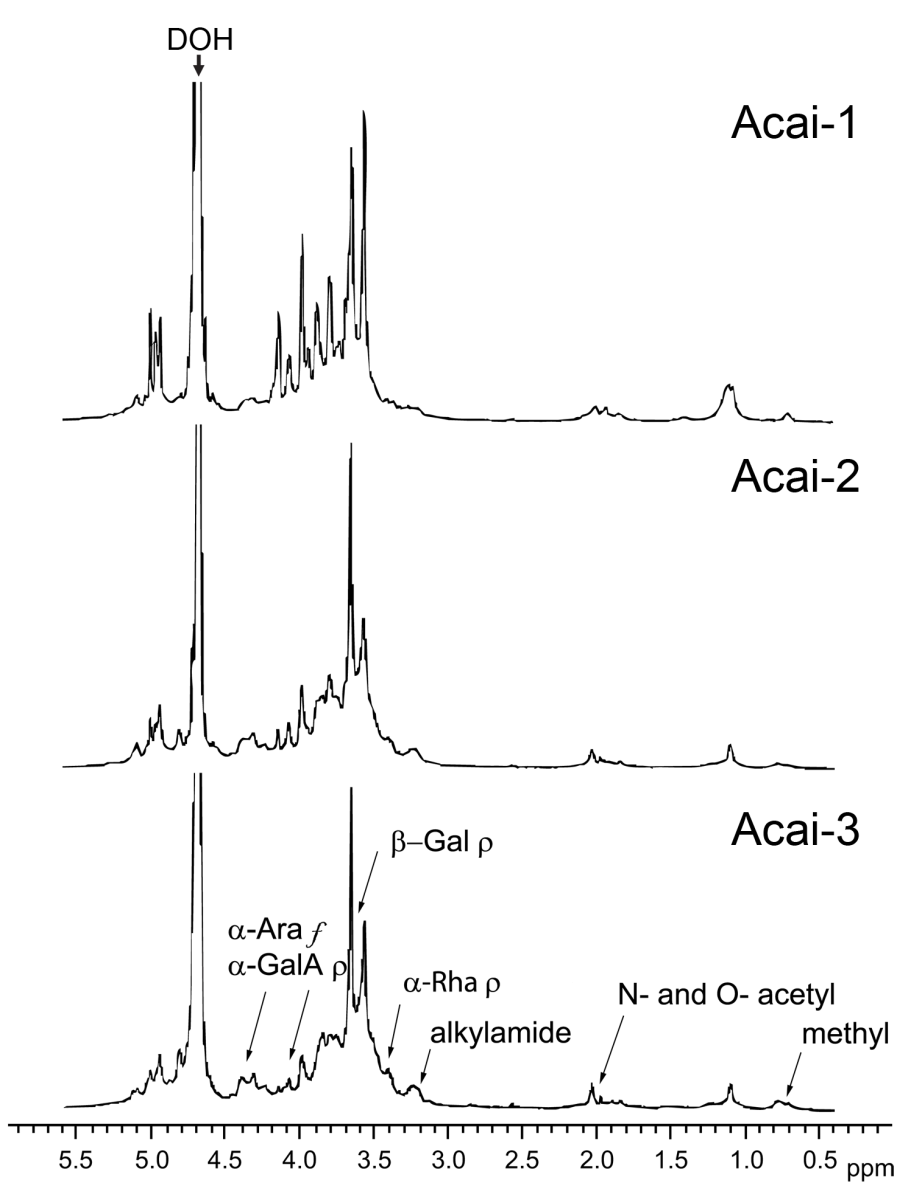


**Supplementary Figure 1** ^1^H NMR spectra of Acai-PS fractions. The fractions were dissolved in D_2_O, and spectra were recorded at 20^o^C, as described [41]. Using previously described methods, we predict the following peak associations which are marked on the Acai-3 graph: weak signals present at 3.37-3.45ppm represent α-rhamnopyranose (α-Rha *p*), the strong signals at 3.54-3.96ppm represent β-galactopyranose (β-Gal *p*) [81], and the signals at 4.04-5.07ppm represent α-arabinofuranose (α-Ara *f*) as well as α-galacturonopyranose (α-GalA *p*) residues [56,81]. N- and O-acetyl (1.9–2.0ppm), methyl (0.75 and 1.1ppm), and alkylamide (3.21ppm) groups are also represented.
